# Analyzing public opinion on China’s “Double Reduction” policy: A sentiment and content analysis of microblog discourse

**DOI:** 10.1371/journal.pone.0338541

**Published:** 2026-03-11

**Authors:** Shuhong Liu, Bo Feng

**Affiliations:** 1 Criminal Investigation Police University of China, Drug Control and Public Security Academy, China; 2 Jilin University, Jilin Province, China; Universita degli Studi di Milano, ITALY

## Abstract

The “Double reduction” policy in China was promulgated and implemented on July 24, 2021. This was the most rigorous and radical education reform since China was founded, aimed at easing pupils’ academic burden and improving their physical and mental health. It sparked extensive public debate across stakeholders (schools, teachers, parents). To delve into public sentiment and improve policy management, this paper analyzes Sina Weibo (Chinese Twitter) posts from July to November 2021. We apply text mining to collect microblogs and comments, and we use Latent Dirichlet Allocation (LDA) for topic extraction. A natural language processing-based (NLP) approach is used to analyze the sentiment. Specifically, we fine-tune a pre-trained Chinese BERT model to classify sentiment, leveraging its superior performance on Weibo data. We report sentiment polarity (positive/negative/neutral) and track its trends over time. We also incorporate engagement metadata (likes/reposts) as indicators. Key findings include the distribution of sentiments, major public concerns, and how sentiment levels evolved around key dates. These insights can help policymakers understand public reactions and guide more responsive education reforms.

## 1. Introduction

Education, as a cornerstone of societal progress and national development, has always been a focal point of public policy and academic research [1]. The importance of education in shaping the future of a nation cannot be overstated, as it directly influences the physical, mental, and intellectual well-being of its citizens [2]. In China, the evolution of the education system has been marked by a series of reforms aimed at addressing the multifaceted challenges faced by students, particularly those in primary and secondary education. Since the establishment of the People’s Republic of China, the basic education sector has undergone five significant reforms, each targeting the physical and mental health of students in compulsory education. These reforms occurred in 1955, 2000, 2013, 2018, and most recently in 2021 (Ministry of Education of the People’s Republic of China, 2021).

The 2021 reform, often referred to as the “double reduction” policy, is arguably the most radical and stringent of these reforms. It fundamentally centers on alleviating the academic burden on students, improving their physical fitness, and reducing the pressure exerted by excessive homework and off-campus tutoring [3]. This policy has evoked significant repercussions among various stakeholders, including schools, teachers, parents, and society at large. The reform has sparked widespread debate and discussion, particularly on social media platforms like Weibo, where the public has expressed a range of emotions and opinions regarding its implementation and potential impact [4].

The integration of public opinion and sentiment analysis has become increasingly important in understanding the societal impact of such policies. Sentiment analysis, a technique widely used in computational linguistics and social sciences, allows researchers to gauge public emotion and identify key concerns related to policy changes [5]. In the context of China’s “ease education burden” policy, sentiment analysis can provide valuable insights into how the public perceives the reform, what their primary concerns are, and how these perceptions evolve over time [6]. Li used big-data methods to analyze media coverage and Weibo discourse, highlighting key public opinion trends.

The purpose of this study is to delve deeper into the public’s reaction to the 2021 education reform by analyzing microblog data from Weibo. Specifically, we aim to answer the following research questions: How positive or negative did the Chinese public feel about the new education policy? What were their utmost concerns regarding the reform? By addressing these questions, we hope to contribute to a more nuanced understanding of public sentiment towards education policies and provide actionable insights for future reforms.

The findings of this study will not only shed light on the public’s perception of the 2021 education reform but also offer valuable recommendations for policymakers. Understanding the emotional and cognitive responses of the public can help in designing more effective and inclusive education policies that address the real needs and concerns of students, parents, and educators [7]. Moreover, this study will contribute to the growing body of literature on sentiment analysis in the context of education policy, offering a methodological framework that can be applied to other policy domains [8].

In conclusion, this study seeks to bridge the gap between public sentiment and education policy by leveraging the power of social media data. By analyzing the emotional and cognitive responses of the Chinese public to the 2021 education reform, we aim to provide a comprehensive understanding of the societal impact of this policy and offer thoughtful recommendations for future reforms.

## 2. Background

### Widely use of social media

The Internet has transformed the way that public opinion was disseminated, allowing everyone to share their views and become producers of information on the Internet, regardless of time and space constraints. While offline social activities are blocked, online social platforms are becoming more and more popular. Internet social platforms play an important role as a part of information exchange.

The online segment of politics is widely perceived to be vital. In the Arab Spring movement, YouTube, Facebook, and Twitter played an important role [9]. Barack Obama is credited as the first politician who uses the web effectively to achieve traditional political purposes [10]. The study of online public opinion through the use of big data can assist government departments with learning about people’s concerns and emotions so that they can respond and take countermeasures in a timely and effective manner to avoid the accumulation of negative emotions that probably have a bad effect on social order. Public opinions can be utilized as an information source to track the effect of policies on society.

### Common problems existing in Chinese young students’ physical fitness

Over these years in China, the “score-only target”, “education involution”, “capitalized education” and “exam-oriented education” have emerged in the education field deeply. It has caused both physical and mental exhaustion of parents and children, the imbalance of the education ecosystem, and also seriously hindered the physical fitness of young people. On September 8th, 2021, the Ministry of Education system held Education Golden Autumn Press Conference to summarize the problems in the school physical education aspect and announce related data. It showed that physical education courses in 22% of nationwide schools were not enough and physical education classes were always being occupied by other academic courses. In 2019, the rate of students aged 6–22 years old achieving excellent physical fitness standards is only 23.8%, which showed the urgency in improving young people’s physical fitness.

### Proposal of “Double reduction policy”

On July 24th, 2021, Chinese authorities published a guideline to ease the burden of excessive homework and academic extramural classes for primary and junior high school students, which is called “Double reduction policy” as the abbreviation. The Ministry of Education is in essence promoting active participation in sports. The “Double reduction policy” on the one hand reduces the burden of homework while freeing up more time and opportunities for students to participate in outdoor activities and physical exercise, and on the other hand classifies sports training as non-academic tutoring, essentially encouraging students to attend extracurricular training in sports.

As for the “Double reduction policy” policy, on the one hand, it requires reducing the homework load and adding after-class services provided by schools, on the other hand, it restricts the academic extramural classes. In detail, homework is not allowed aiming to the children below the grade 3 and homework for junior high school students is limited to less than 90 minutes to finish. Due to the guideline, Extra-curricular classes where the tutors teach advanced academic content of the school curriculum are forbidden. According to Global Times, over 75% of Chinese urban children, from first grade to twelfth grade, are enrolled in extramural classes including academic tutoring and interest tutoring. However, some tutoring institutions take parents’ anxiety as a marketing strategy, selling unnecessary tutoring programs which make the burden on students increase. This phenomenon in education has resulted in many social problems including poor physical fitness of pupil, more psychological problems, and excessive family expenditure.

### Hot trends of “Double reduction policy”

**Fig 1:**
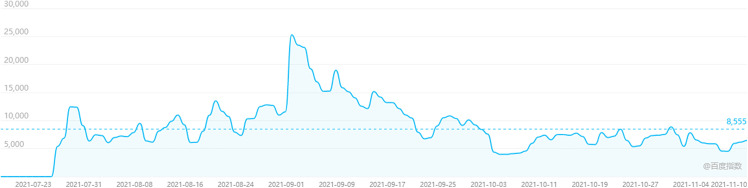
Baidu search index curve of “Double reduction policy”.

The publication of the policy has aroused widespread concern among citizens. The Baidu Search Index is based on the search volume of internet users in Baidu and uses keywords as the statistical object to scientifically analyze and calculate the weighted sum of each keyword’s search frequency in the Baidu web search platform. It is one of the most vital statistical analysis platforms in China’s internet currently and even the whole data era. Searching for the keyword “Double reduction policy” through the Baidu index, the search index in Fig 1 shows that after the policy was released on July 24th, 2021, searching times steeply rise to 5404 on that day. In general, it fluctuated over the three months. The peak reached 25305 on September 1^st^ which was the new start of the fall semester in China and also the date of the policy came into effect.

According to the microblog content crawled from policy publishing date to November 10^th^, 2021, the first few days focused on the release of the “Ease Education Burden Policy” and the Ministry of Education of the People’s Republic of China Q&A interview on the Policy, followed by microblog on the details of supporting measures a few days later. Therefore, the discussion about physical education brought by the policy also increased significantly after people started focusing on supporting measures.

## 3. Methodology

According to the discourse expressed in posts on a particular topic, sentiment can be perceived as positive, neutral, or negative. Sentiment analysis has been utilized to analyze customers reviews on social media, events [11], politics [12,13], construction projects [14,15], education [1], etc. It is necessary to understand the words and categorize sentiments into their groups when sentiment analysis is performed [16]. Sentiment analysis lies between NLP and natural language understanding.

SnowNLP is a Chinese natural language processing Python library based on TextBlob. It can perform sentiment analysis of text based on its processing capabilities for Chinese text. SnowNLP technique, as well as a dictionary of stop words constructed by the author, were applied to score each text for the sentiment. The closer the scoring value is to 1, the more positive it is. Likewise the closer it is to 0, the more negative it is. After the scoring was completed, the scores were analyzed and it was found that texts with scores higher than 0.67 showed positive emotions and those with scores less than 0.33 showed negative emotions. Texts between 0.33 and 0.67 showed a neutral attitude. After the classification was completed, a manual test was conducted.

To gain a deeper understanding of public sentiment and the underlying themes related to China’s 2021 education reform, this study employs a two-step analytical framework: sentiment analysis followed by topic modeling using Latent Dirichlet Allocation (LDA). The methodology is designed to extract both the emotional tone and specific concerns expressed in microblog data from Weibo. The first step in the analysis involves classifying the sentiment of microblog posts into positive, negative, or neutral categories. Sentiment analysis is conducted using a pre-trained model fine-tuned for Chinese text, which leverages a combination of lexical features and machine learning algorithms to accurately classify emotions. The model is validated on a labeled dataset of Weibo posts to ensure its reliability [17]. This step provides a high-level overview of the public’s emotional response to the education reform, allowing us to quantify the proportion of positive, negative, and neutral sentiments. While sentiment analysis offers valuable insights into the emotional tone of public discourse, it does not reveal the specific themes or concerns driving these emotions. To address this limitation, LDA was employed, a probabilistic topic modeling technique, to extract latent topics from the text data. LDA, introduced by, is a three-layer Bayesian model that assumes documents are mixtures of topics and topics are distributions over words. It is particularly well-suited for this study due to its ability to uncover hidden thematic structures in large, unstructured text datasets without requiring labeled data [18].

The LDA process begins with preprocessing the text data. Topic modeling was implemented using Gensim’s LDA library. We experimented with topic counts (K = 2–15) and selected the optimal number based on the highest coherence score (CV metric) while maintaining interpretability. To verify stability, we ran the model with 10 random seeds and observed a 92% topic-word overlap across runs. Raw microblog posts are cleaned by removing stop words, punctuation, and non-relevant characters. Tokenization and lemmatization are then performed to standardize the text. The preprocessed data is converted into a document-term matrix, which serves as the input for the LDA algorithm. The number of topics is determined through a combination of domain knowledge and coherence score optimization, ensuring that the extracted topics are both interpretable and meaningful [19].

Once the LDA model is trained, it outputs two key distributions: the distribution of topics for each document and the distribution of words for each topic. Each topic is represented as a list of words with associated probabilities, which are interpreted to identify the underlying themes. For example, a topic characterized by words such as “homework,” “pressure,” and “tutoring” might be labeled as “Academic Burden.” These topics are then linked to the sentiment classifications to explore how specific themes correlate with positive, negative, or neutral emotions.

**Fig 2:**
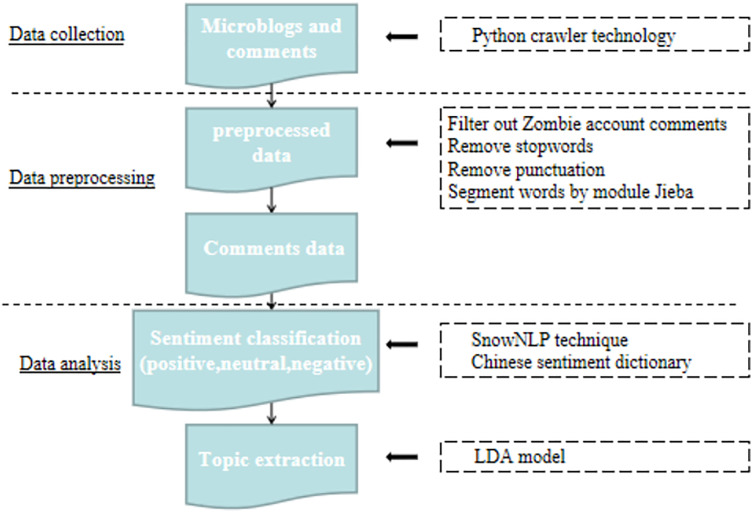
Overall analytical framework of the method.

The final step in the methodology involves integrating the results of sentiment analysis and topic modeling. By mapping the extracted topics to their corresponding sentiment classifications, we can identify which themes are most strongly associated with positive or negative emotions. For instance, if negative sentiment is predominantly linked to topics such as “Academic Stress” or “Inequality in Education,” this suggests that these issues are key drivers of public dissatisfaction with the reform. Conversely, positive sentiment associated with topics like “Physical Fitness” or “Reduced Homework” highlights aspects of the reform that are well-received by the public. To ensure the robustness of our findings, we validate the LDA model using a combination of qualitative and quantitative methods. Topic coherence scores are calculated to assess the interpretability of the extracted topics, while manual inspection of topic-word distributions is conducted to verify their relevance to the education reform context. Additionally, the sentiment analysis results are cross-checked against a sample of manually annotated posts to confirm the accuracy of the classification. The study adheres to ethical guidelines for social media research. All data is anonymized to protect user privacy, and only publicly available posts are analyzed. The research is conducted in compliance with Weibo’s terms of service and relevant data protection regulations. In conclusion, the combination of sentiment analysis and LDA-based topic modeling provides a comprehensive framework for analyzing public opinion on China’s 2021 education reform. This approach not only quantifies the emotional tone of public discourse but also uncovers the specific concerns and themes driving these emotions, offering valuable insights for policymakers and researchers alike (See Fig 2).

## 4. Data analysis

### Data collection

In China, there are a few social media platforms such as WeChat, Sina microblog, QQ with millions of end-users. Sina microblog is chosen in this study as the social media platform for raw data collection because it is a hugely popular platform known for its traits of instant messaging, transparent sharing, and public accessibility [15].In the data mining phase, the sample data for this study was obtained from 14 official Sina microbloggers including 11 national-level official microbloggers and 3 influential media microblogers, named by People’s Daily (142 million), CCTV News (119 million), Xinhua News Agency (104 million), People’s Daily (79.42 million), China News (71.74 million) and China Daily (6,176 million), Headline News (104 million), Sina News (62 million), Daily Economic News(48 million), Pengpai News (30 million), Sina Technology (24 million), Fenghuang Weekly News (22 million), Guangzhou Daily (19 million), China Youth News (19 million). With Scrapy open-source framework developed in the Python language, the crawler program was applied to browse the Sina microblog website to obtain online data pertinent to “Double reduction policy” and “Physical fitness” with the specified time frame (i.e., from July to November 2021). We collected Weibo posts related to “Double Reduction” between July 24 and November 10, 2021. Specifically, we crawled official microblogs from education authorities and relevant hashtags, yielding 15 primary posts with their comments.

The focus on official accounts was intentional, as the study aimed to analyze how the public reacts to authoritative or policy-related information sources. However, this sampling frame introduces platform and topical bias, since audiences engaging with official accounts may not represent the wider Weibo population. To address this, we conducted sensitivity checks by comparing sentiment distributions across different posts and by re-sampling subsets of comments. The results showed consistent sentiment patterns, indicating that the findings are robust within this communication context.

### Data preprocessing

Data preprocessing is required before data analysis because the data from the crawling approach is incomplete and inconsistent. The process in detail was in the following text.

The relevant data were firstly processed using the MS Excel application [20]. A second-round data cleaning was conducted to filter out the comments by zombie accounts (automated accounts to inflate follower counts). All user details were removed to ensure anonymity and unbiased analysis. Then the Chinese word segmentation module Jieba from Python was used to segment every data, filter deactivation, eliminate stop words in preparation for the following topic and sentiment analyses. After initial cleaning (removing duplicates and irrelevant content), the dataset comprised 15 microblogs and 56,080 comments. Each comment record includes: post content, comment text, timestamp, number of likes, and repost count. (For privacy, user IDs were removed.) (Table 1).

**Table 1:** Contrast between raw data and preprocessed data (translated from Chinese).

Raw data	Preprocessed data
WujianxingzheNo1:As parents, it is impossible not to be anxious when the College Entrance Examination doesn’t change.[facepalm][facepalm]	As parents, it is impossible not to be anxious when the College Entrance Examination doesn’t change.

Preprocessing steps included tokenizing Chinese text using Jieba, removing stop words and punctuation, and mapping synonyms to a standard form. Importantly, we retain emojis and emoticons in the text, since Chinese microblog emoticons carry sentiment. These symbols are encoded as tokens so that our model can learn from their usage. For example, “[facepalm]” expresses frustration and is treated as part of the comment content. We justify this inclusion because previous research has shown Weibo emoticons contribute meaningfully to sentiment. All text preprocessing and modeling were conducted on the original Chinese text. English translations of topic labels and representative comments were produced only after modeling, for reporting purposes. Two bilingual researchers independently translated topic keywords and exemplar comments, followed by back-translation checks to ensure semantic consistency.

## 5. Result

### Sentiment analysis

We use Chinese BERT pre-trained model, which has demonstrated high accuracy on Weibo sentiment tasks. The model is fine-tuned on a labeled Chinese microblog sentiment dataset. Comments are tokenized (including emojis), and fed into the model to obtain sentiment probabilities. We classify each comment as positive, negative, or neutral by taking the highest-probability class. To validate the model, we performed a manual annotation. Two researchers independently labeled a random sample into the three sentiment classes. Inter-annotator agreement was about 94.2%, indicating reliable coding. Disagreements were resolved by a third coder. These labels are used to test model accuracy, which reached 85% on held-out data.

In this study, sentiment polarity refers strictly to the emotional tone (positive/negative/neutral) of the comment. We treat it separately from stance. For example, a comment may exhibit negative sentiment (e.g., worry or criticism) but still express overall support for the policy’s goals. This distinction is noted to avoid confusion. Analysis of citizen comments’ sentiment showed that negative and neutral sentiment occupied a huge part in the comments, indicating that citizens had more reserved opinions about the official microblog content with relevant policies. Specifically, in 5608 comments from citizens, there were 1671 negative comments, accounting for 29.8%; 1494 neutral comments, accounting for 32.0%; and 1962 positive comments, accounting for 35.2%. We emphasize that this balance means neither overwhelmingly positive nor negative sentiment dominated. In the discussion we interpret this nuance in depth. For now, we note quantitatively: the slight excess of positive posts suggests some optimism (e.g., support for reducing homework), while the significant negative fraction highlights concerns (e.g., about implementation). Neutral comments (around one-third) often contained informational queries or complex reflections.

### Topic analysis

**Fig 3:**
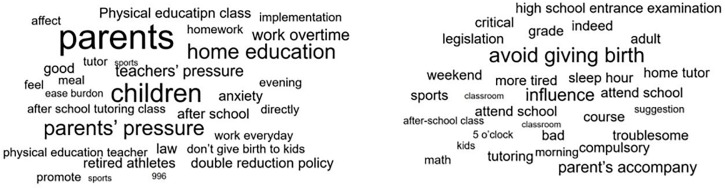
Word cloud analysis of microblogs’ comments.

To further understand the logic behind citizens’ emotions, this paper has extracted keywords from citizens’ comments. Keywords that were not relevant to this study were excluded and close equivalent expressions were combined. Then a word cloud analysis of microblogs’ comments is created as Fig 3 shows. A word cloud is a form of text data visualization in which tags are single words. The relative sizes of words represent their weighting or importance in the context of the text considered [12]. The most prominent words in the word cloud in Fig 3 are “children” (孩子), “parents” (家长) “home education” (家庭教育) “physical education teachers” (体育老师) “athlete” (运动员) “retired” (退役) “work overtime” (加班) “pressure” (压力) “teacher” (老师) “implement” (落实) “after school tutoring class” (托管) “measures” (配套措施) “anxiety” (焦虑) “tutoring” (辅导). Hot words above indicate that the support measures in home-school-society three parties were the most concerning issues by the public.

a.Word cloud of word frequency rank 1–50 b. Word cloud of word frequency rank 50–100

To find out the root causes of different emotions of internet users, the topics based on the three emotions as categories were extracted. The top 8 tokens from each topic were used to represent the topic. Table 2 lists the word-level topics as identified by LDA.

**Table 2:** Topics generated by LDA analysis.

Sentiment	Topic	Terms under each sentiment
Positive	1	physical fitness, good, double reduction policy, improve, support, homework, sports, health
	2	retired, professional, athletes, idol, double reduction policy, opportunity, sports, teacher
	3	double reduction policy, home education, parents, teacher, children, support, precious, time
Negative	1	academic grade, implement, double reduction policy, follow-up, after school, tutoring, delay, home education, accompanied
	2	double reduction policy, extra-curricular class, family, cost, sports, art, income, children,
	3	National College Entrance Examination, academic course, double reduction policy, grade orientation, physical education, parents, pressure, children
Neutral	1	implement, double reduction policy, physical exercise, equipment, homework, district, basic education, school tutoring, healthy
	2	guidance, cooperation, double reduction policy, academic grade, home education, school tutoring, motivation, communication

### Negative attitude

From the content of the comments corresponding to negative sentiments, citizens’ topics can be summarized into 3 categories.

Firstly, whether supporting measures can be implemented successfully is the biggest concern. The policy has just been introduced and many follow-up measures need to be added. Some comments are like, “Due to after-school tutoring, kids arrive at home so late which causes they have to delay dinner time. “, “previously what was not learned well in school could be further consolidated in tutoring classes out of school.”, “Only two hours spent with parents a day is too little”, “More sports and art tutoring, less homework for kids at school but how about their academic courses grade?”, “After canceling tutoring institution for academic courses, it seems that no one can teach her except her teacher in school because both we parents are not well-educated.”, “what my son would do after school if both parents have no time to be with him. “, “kids have more time with electronic devices”Parents are concerned about whether more flexible supporting measures. For example, dinner at school could be arranged to adapt to the new regular school time.

Secondly, extra-curricular sports training classes cost parents a lot. Some comments are like, “From mathematics and English these extra-curricular tutoring transferring to sports, art, music-related ones cost more”, “It is more advantageous to develop children’s comprehensive quality with a high family income.”,”more kinds of sports in school are expected. As well known, most parents with children who participate in sports are well aware of the financial costs associated with their child’s involvement, with studies suggesting that the costs of sport participation are among the most important barriers to children’s participation in sport, especially among low-income parents [21,22]. Theoretical explanations for this relationship have been offered by [23].”Unequal distribution of opportunities between different social-economic status families suggests that extra-curricular sports training is more like the ‘preserve’ of the wealth especially high-spending sports, e.g., golf, skiing. citizens call for regulation on extramural classes fees. At the same time, more sorts of sports are advised to introduce into on-campus courses, which could lead to fewer extracurricular needs.

The third topic is the discussion concerning the National College Entrance Examination system. One root cause of China’s education involution is the National College Entrance Examination system. Comments are like“If this exam only focuses on academic course grade, the pressure still exists.”, “whether the test on physical education could be involved in National College Entrance Examination grade?”.”After all, all stages of a child’s development are still dependent on academic performance, and I dare not slacken off in the foundation stage currently.”

### Neutral attitude

Concerning the neutral sentiment, there are mainly 2 categories.

Firstly, the extent of implementation is the key point. People comments like“rural area and urban area both process enough physical exercise equipment.”, “The key to a good policy is to implement it well. School tutoring is indeed for children’s interests and sports instead of writing homework in another way.”,” To truly serve the modern education system and to protect the healthy development of basic education in China.” Constructing equity in education and reducing the gap is a permanent goal for China. More supporting measures and regulatory instruments need to be put in place.

The other topic is that parents are more proactive in cooperating with the schools.“ We must be conscious of the fact that easing the burden of homework cannot reduce the children’s motivation to study, and that canceling academic extra-curricular tutoring doesn’t represent weakening home supervision”.”In this case, our children need more guidance from us parents than before.”,”With extra-curricular institutions withdrawing from academic tutoring and schools weakening students’ homework at the same time, parents play a greater role in children’s growth and academic course study”.”Parents need to fulfill the primary responsibility for home education. “On the one hand, physical exercise is emphasized by parents, on the other hand, parents need to enhance communication with teachers and provide academic course tutoring for their kids in case.

### Positive attitude

As for the positive attitude toward the policy, the topics are classified into 3 categories.

Firstly, developing students’ physical fitness got broad support. Comments are as followed. “Due to the stricter regulations on academic tutoring classes, many parents like me choose sports or another tutoring for developing interests instead. It’s quite good for kid’s health.”,”We hope that our children can get more physical exercise out of school and develop hobbies.”Many parents attach importance to children’s physical exercise.

Secondly, retired professional athletes are highly encouraged to enter school as physical education teachers. Parents said that well-known athletes were professional and also super idols for children. They could set a good example for young students. Inviting retired excellent athletes into schools solves the problem of lacking sports teachers on the one hand, and provides a good career opportunity for those excellent athletes who retired at a young age on the other hand.

Last, part of parents’ support for family education. They said as followed, “It has been a long time that students were just between schools and extra-curricular classes, it’s time for family education.”,”parents are the best teachers for their children.”

## 6. Conclusions and implications

### Conclusions

The analysis of public sentiment on the “Double Reduction” policy reveals that while negative, neutral, and positive sentiments share a common concern for more detailed supporting measures, they differ in focus. Negative sentiments primarily highlight issues such as the cost of sports and arts tutoring, children’s academic performance, and the National College Entrance Examination system. Neutral sentiments center on the policy’s thorough implementation and the importance of home-school cooperation. Positive sentiments, on the other hand, emphasize the policy’s potential to reduce academic burden and promote holistic development. Online public opinion analysis not only tracks changes in public sentiment but also offers actionable insights for governments and media to guide public opinion. The “Double Reduction” policy is widely supported for its aims, but its success hinges on addressing implementation gaps and fostering collaboration among families, schools, and the government. Government Measures: To alleviate parental anxiety, the government must ensure strict regulation and supervision of policy implementation. This includes providing flexible school schedules, after-school meals, and subsidies for high-cost sports programs. Additionally, reforming the National College Entrance Examination system and emphasizing process-based assessments in physical education can help reduce the overemphasis on academic outcomes. Schools should prioritize physical fitness and expand sports programs to bridge inequality gaps caused by family economic status. Offering diverse sports activities, inviting retired athletes as coaches, and improving after-school services can enhance students’ overall development. Parents must shift traditional attitudes and actively support their children’s physical and artistic pursuits. Strengthening family education, which has long been neglected, is crucial for the policy’s success.

In conclusion, the “Double Reduction” policy holds significant promise, but its effectiveness depends on coordinated efforts from all stakeholders to address existing challenges and ensure its holistic implementation.

### Implications

How the relevant public opinion reacts has not only a yardstick significance for the decision making but also a feedback significance for the implementation of education policies, as well as an ontological value for the resolution of educational conflicts. The findings have great implications for different stakeholders.

### Limitation

We acknowledge several limitations. First, because our dataset was restricted to comments on 15 official microblogs, it primarily reflects responses to authoritative discourse rather than general user-generated posts. Future work should expand the sampling frame to include non-official sources to improve generalizability. Second, Weibo does not provide reliable demographic attributes (e.g., age, parental status, education level), so we were unable to examine sentiment differences across demographic groups. Only basic metadata such as declared location and verification status were available and used for descriptive statistics. Future research could combine survey-linked or inferred demographic data to explore these differences, as demonstrated in prior studies

## Supporting information

S1 File Data. (ZIP)
